# Improved R-wave detection for enhanced cardiac Gating using an MRI-compatible 12-lead ECG and multi-channel analysis

**DOI:** 10.1186/1532-429X-13-S1-P3

**Published:** 2011-02-02

**Authors:** Zion Tsz Ho Tse, Charles L Dumoulin, Gari Clifford, Michael Jerosch-Herold, Daniel Kacher, Raymond Kwong, William Gregory Stevenson, Ehud Jeruham Schmidt

**Affiliations:** 1 Brigham and Women's Hospital, Boston, MA, USA; 2 University of Cincinnati College of Medicine, Cincinnati, OH, USA; 3 University of Oxford, Oxford, UK

## Background

An important requirement for successful cardiac MRI is accurate gating. However, obtaining proper Electrocardiogram (ECG) gating inside the MRI is a difficult problem, due to the Magneto-Hydro-Dynamic (MHD) effect, resulting in frequent intermittent gating. The MHD effect generates a voltage due to conductive blood flow perpendicular to B_0_, and distorts the real ECG. Intermittent gating also occurs when arrhythmia patients incur several events of arrhythmia between their sinus rhythm (SR) beats, resulting in blurred images. We hypothesized that a novel real-time 3-D gating method could accurately detect the QRS complex, even in difficult cases, such as with Premature Ventricular Contractions (PVCS), non-stable heart rate (atrial fibrillation, exercise), imaged at both 1.5 and 3T MRIs.

## Methods

**Figure 1:**
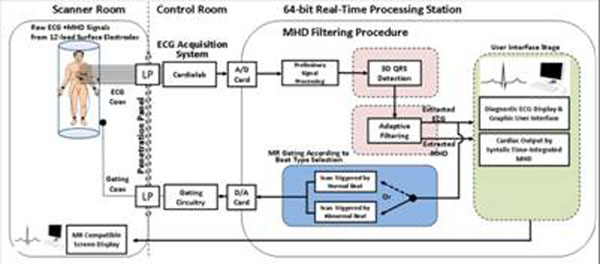
12-lead ECG system with real-time output for scan triggering.

**Figure 2:**
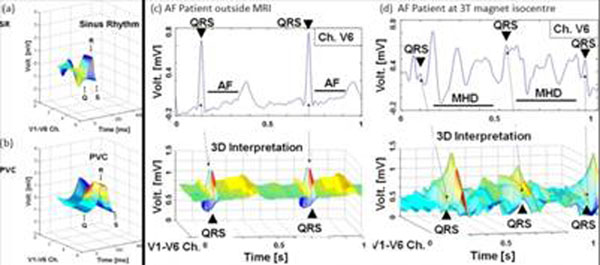
3-D representation of ECG signals: PVS pateint’s (a) and SR (b) PVC beats taken outside a 3T. AF patient’s ECG (c) outside and (d) inside a 3T: Channel V6 signal (top) and 3D representation (bottom). 3D-QRS is identifiable even with stronger MHD peak.

Fig. 1 shows our ECG system [1]. A gating method based on a 3-D ECG representation is developed. By making ECG channels V1-V6 represent a third axis, an additional dimension is added to the time and voltage axes. The channel axis (Fig. 2(a-b)) carries extra information on the electrical signal propagating from the source in the heart to the surface leads. Within this, the QRS complex forms a unique 3-D geometry, which is identifiable even with a large MHD effect using a fast FFT 2-D cross-correlation subroutine to achieve a real-time computation speed of <5msec.

## Results

**Figure 3:**
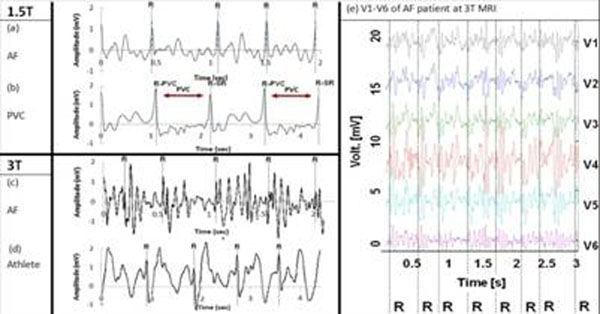
(a-d) 20-sec breath-held ECGs obtained at 1.5T (a,b) and 3T (c,d) MRIs for AF, PVC and exercising athlete subjects. R designates detected R-wave peaks using 3D-QRS. (e) V1-V6 traces of the AF patient at 3T. All R peaks (dotted lines) are correctly identified (a-e).

Fig. 2(a-b) shows the 3-D QRS kernels of SR and PVC beats. The PVC kernel (b) is significantly different from the SR (a) not only along the time axis but also the channel axis. Because PVC foci are located at ventricular locations, source location and electrical wave propagation to the surface electrodes are different, which affect the channels axis, providing a unique 3-D geometry distinguishable from the SR kernel. Fig. 2(d) shows ECGs of an Atrial Fibrillation (AF) patient taken inside a 3T, in which the QRS complex is hardly identified in V6 (top), smaller than the MHD signal. However, the SR QRS kernels (bottom) are distinguishable with their unique 3-D features in Fig. 2(c-d), since the MHD originates from the aortic arch and SR from sinus node. Fig. 3 shows the gating results of 2 AF and 1 PVC patient, as well as 1 athlete subject exercising to produce heart rate variation from 44bpm to 87bpm.

## Conclusions

The gating method, based on a unique 3-D ECG representation, allows accurate R-wave detection and separation of beat types even with strong MHD signals.
